# Attention

**Published:** 1914-10

**Authors:** 


### Attention.

I wish to call your attention to the "want advertising department." If you have anything to sell, or if you want to buy anything, just send your advertisement for the same along to the editor. Look over your instrument case, your books, and office fixtures and see if there isn't something there that you are not using, that someone else would be glad to pay a reasonable price for, and remember when you put a better instrument in the hands of a brother physician, in fact, when you help him in any way, you have elevated the entire profession to just the degree that you helped him.

